# Epidemiology and treatment of children with hereditary angioedema in Germany: A retrospective database study

**DOI:** 10.1002/clt2.12313

**Published:** 2023-11-13

**Authors:** Freerk Prenzel, Susanne Abraham, Christoph Hirche, Gerrit Müller, Stephan Kaiser, Leonarda Serdani‐Neuhaus, Rebecca Zingel, Inmaculada Martinez‐Saguer

**Affiliations:** ^1^ Department of Pediatrics University of Leipzig Medical Center Leipzig Germany; ^2^ Department of Dermatology University Allergy Center University Hospital Carl Gustav Carus TU Dresden Germany; ^3^ Takeda Pharma Vertrieb GmbH & Co. KG Berlin Germany; ^4^ Epidemiology IQVIA Frankfurt Am Main Germany; ^5^ Pediatrics Haemophilia Centre Rhine Main (HZRM) Moerfelden‐Walldorf Germany

**Keywords:** Germany, HAE attack, hereditary angioedema, long‐term prophylaxis, pediatric population

## Abstract

**Background:**

Hereditary angioedema (HAE) is a potentially life‐threatening inherited disease that causes recurrent, serious, and debilitating episodes of swelling. While evidence has improved in adult patients, data on the epidemiology and treatment of pediatric patients with HAE remain very limited. The aim of this study was to determine the incidence and prevalence of pediatric patients with HAE aged <12 years, as well as treatment patterns, co‐medication, and specialties involved.

**Methods:**

In this retrospective study (2016–2021), the German IQVIA^TM^ pharmacy claims (LRx) database was used to analyze prescriptions of HAE‐specific treatments and co‐medications.

**Results:**

We found an HAE prevalence in pediatric patients aged <12 years of 2.51:100,000 and a 12‐month prevalence of up to 1.02:100,000 between 2016 and 2021. Most HAE treatments were prescribed by outpatient clinics and pediatricians, with an increasing proportion of icatibant as an on‐demand treatment and low rates of long‐term prophylaxis (LTP). The prescription rate of analgesics as the most common co‐medication decreased notably after HAE diagnosis.

**Conclusion:**

Our findings provide insights into the epidemiology and current pediatric HAE treatment landscape in Germany. The obtained HAE prevalence in pediatric patients aged <12 years was even higher than the previously reported average of overall cohorts, whereas the LTP rate was low, which might indicate an unmet need for newer LTP treatment options in pediatric patients.

## 1 KEY MESSAGE

This study sheds light on the limited data regarding the epidemiology and treatment of pediatric patients with hereditary angioedema (HAE). The findings reveal a higher prevalence of HAE in children <12 years of age compared with previously reported data, together with low rates of long‐term prophylaxis (LTP). These results highlight the need for further research and development of improved treatment options for pediatric HAE patients.

## 2 BACKGROUND

Hereditary angioedema (HAE) is an autosomal dominant genetic disease that can cause serious and debilitating attacks of swelling in the abdomen, face, feet, genitals, hands and throat. Besides the visible and painful swelling of the skin other symptoms may include nausea, abdominal pain and/or diarrhea, and even asphyxiation due to laryngeal edema, rendering the disease a potentially life‐threatening condition.^1^ HAE most commonly results from mutations in the SERPING‐1 gene resulting in a deficiency (HAE type 1, 85%) or dysfunction (HAE type 2, 15%) of the C1 esterase inhibitor (C1‐INH). The proportion of de novo mutations is estimated to be 20%–25% and there are no known differences between sexes or ethnicities.^2^, ^3^ Several other mechanisms with normal C1‐INH have been described.^4^

Pathophysiologically, C1‐INH deficiency leads to accumulation of the tissue hormone bradykinin, which causes vascular leakage and edema formation.^4^ The onset of the disease varies but usually occurs in childhood or adolescence.^5^ The reported median age at first attack is 11 years, with a wide age range.^6^ HAE attacks often increase in number and intensity during puberty due to hormonal changes.^3^

When attacks occur, HAE‐patients must receive immediate on‐demand treatment. Current guidelines recommend that patients should be evaluated for the indication of LTP at each visit to ideally achieve attack‐free status, resulting in complete control of the disease and normalization of patients' lives.^3^ Short‐term prophylaxis (STP) is recommended before surgery or medical procedures that may trigger an HAE attack.^3^, ^5^

In Germany, four treatments are approved for the on‐demand treatment of HAE attacks in adults and adolescents, namely plasma‐derived intravenous (i.v.) human C1 inhibitor (pdC1‐INH) I and II, icatibant and conestat alfa (recombinant human C1‐esterase inhibitor).^7^ For the on‐demand treatment of pediatric patients, human pdC1‐INH I (i.v.) is approved starting in infancy, pdC1‐INH II (i.v.), icatibant and conestat alfa are approved for children 2 years of age and older.^7^, ^8^

For LTP, five treatments are available for adults in Germany: pdC1‐INH II (i.v.),^7^ subcutaneous (s.c.) lanadelumab, pdC1‐INH I (s.c.),^9^ berotralstat and tranexamic acid.^7^ For children and adolescents, lanadelumab, pdC1‐INH I (s.c.) and berotralstat are approved as LTP starting at the age of 12 years, and pdC1‐INH II (i.v.) starting at the age of 6 years^8^ For children <12 years, the international HAE guideline recommends treatment with pdC1‐INH (i.v.) or icatibant for acute attacks and administration of pdC1‐INH as STP or LTP.^3^ There are two treatment options for plasma‐derived human C1 esterase inhibitors from two different manufacturers. The first option is pd‐C1INH I, which is available in two application forms: intravenous and subcutaneous. The second option is pd‐C1INH II, which is available in intravenous form only.

While findings from observational registries and surveys, also from Germany, have improved the evidence for adult HAE patients, epidemiological and treatment data for pediatric patients with HAE remain very limited. The aim of this study was to determine the incidence and prevalence of HAE in pediatric patients aged <12 years with statutory health insurance (SHI) and to generate evidence on treatment patterns and treating specialties.

## 3 METHODS

### 3.1 Study design and data source

**FIGURE 1:**
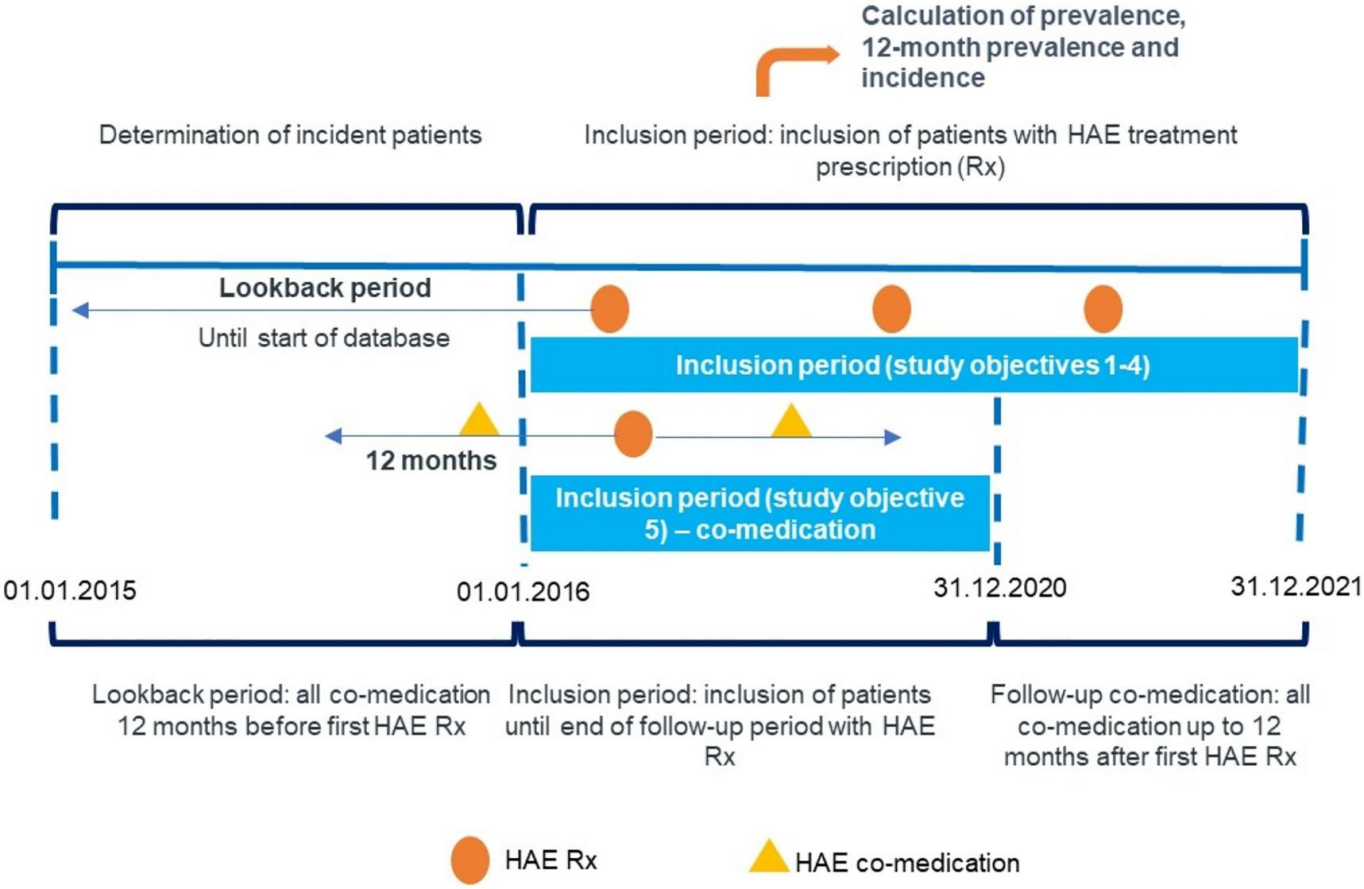
The analyzed patient population consisted of statuary health insurance patients with at least one prescription of HAE treatment (EphMRA‐ATC B06D0) between January 2016 and December 2021 through public pharmacies.

This non‐interventional, retrospective database study describes real‐world utilization patterns in all German patients receiving HAE therapy using the German IQVIA^TM^ pharmacy claims (LRx) database. The analyzed patient population consisted of SHI patients with at least one prescription of HAE treatment (EphMRA‐ATC B06D0) between January 2016 and December 2021 through public pharmacies. A lookback period to January 2015 was used to identify new patients. For the analysis of the co‐medication in pediatric patients, patients aged 0–10 years with at least one HAE treatment between January 2016 and December 2020 were included to ensure a 12‐month baseline and follow‐up period to assess the co‐medication within 1 year after the first HAE treatment (Figure 1).

IQVIA^TM^ LRx is a longitudinal pharmacy administrative database that collects data from retail pharmacy coding centers. The data is primarily used for reimbursement purposes, which allows a longitudinal follow‐up of these patients. The database covers 84% of German SHI patients nationwide which has been shown to be a robust data set in previous studies.^10^ As the dataset was anonymized, no ethical approval and no informed consent of patients was required.

### 3.2 Study outcomes and definitions

In this study between 2016 and 2021, epidemiological data on HAE were obtained and the treating specialties along with the applied treatment were analyzed.

The prevalence, 12‐month prevalence, and annual incidence, along with the proportion of HAE patients with LTP (all ages, 0–11 years, and as an exploratory analysis, 2–11 years) were determined. Long‐term prophylaxis was defined as prescription of pdC1‐INH I (s.c.) or pdC1‐INH II (i.v.) with >7 vials/28 days or any prescription of berotralstat or lanadelumab. Additional on‐demand treatment is always prescribed for patients with LTP, which helps to identify these patients.

Due to insufficient evidence from outdated studies and lack of efficacy, tranexamic acid is not recommended in most guidelines. Therefore, and due to its use in other indications, tranexamic acid was not included in this study. However, additional on‐demand treatment is always prescribed for patients with LTP, and therefore the patients were nevertheless identified in the respective on‐demand analyses.

The different treating specialties of HAE patients after initial prescription were recorded, as well as the type and quantity of HAE treatments in pediatric patients aged 2–11 years, including additional on‐demand treatment under LTP. On‐demand treatment was defined as a prescription of icatibant, conestat alfa, pdC1‐INH I or II (i.v.) with ≤7 vials/28 days. Children <2 years were not included in this analysis because there is no approval and recommendation for LTP at this age.^8^ Average vial consumption of HAE treatments for the age subgroups 2–5 and 6–11 years and co‐medication in pediatric patients aged 0–10 years at first HAE treatment and within 1 year before and after first HAE prescription was analyzed.

### 3.3 Analysis methods

For epidemiology, patient numbers were extrapolated to the SHI level in Germany using the IQVIA^TM^ Pharma‐Scope database. Subsequently, reference data of all SHI patients from the German Federal Health Reporting System were used for the total population and an approximation was made with DESTATIS data for pediatric patients <12 years, respectively (Supplementary Table S1).

The treating specialty for each prescription for HAE patients between 2016 and 2021 was cascaded. If the initial prescription by a specialty was followed by additional prescriptions by another specialty, the patient was cascaded from the first specialty to the second specialty. Missing values were reported as missing; extrapolation was not performed. Descriptive statistics, counts and percentages for categorical variables were calculated. Data analysis was performed using SAS 9.4 (SAS Institute Inc.).

## 4 RESULTS

### 4.1 Epidemiology

In this study, a total of 2436 SHI patients received HAE treatments between 2016 and 2021, representing a prevalence of 3.35:100,000. In children <12 years, who received HAE treatments between 2016 and 2021, the prevalence was 2.51:100,000 persons. There were more female participants in the overall study population (66.2%) and in pediatric patients <12 years (55.5%). There were regional differences, with the highest prevalence in Western Germany (4.35:100,000 overall population, 3.35:100,000 in children <12 years) (Supplementary Figure F1, Supplementary Table S4).

**FIGURE 2:**
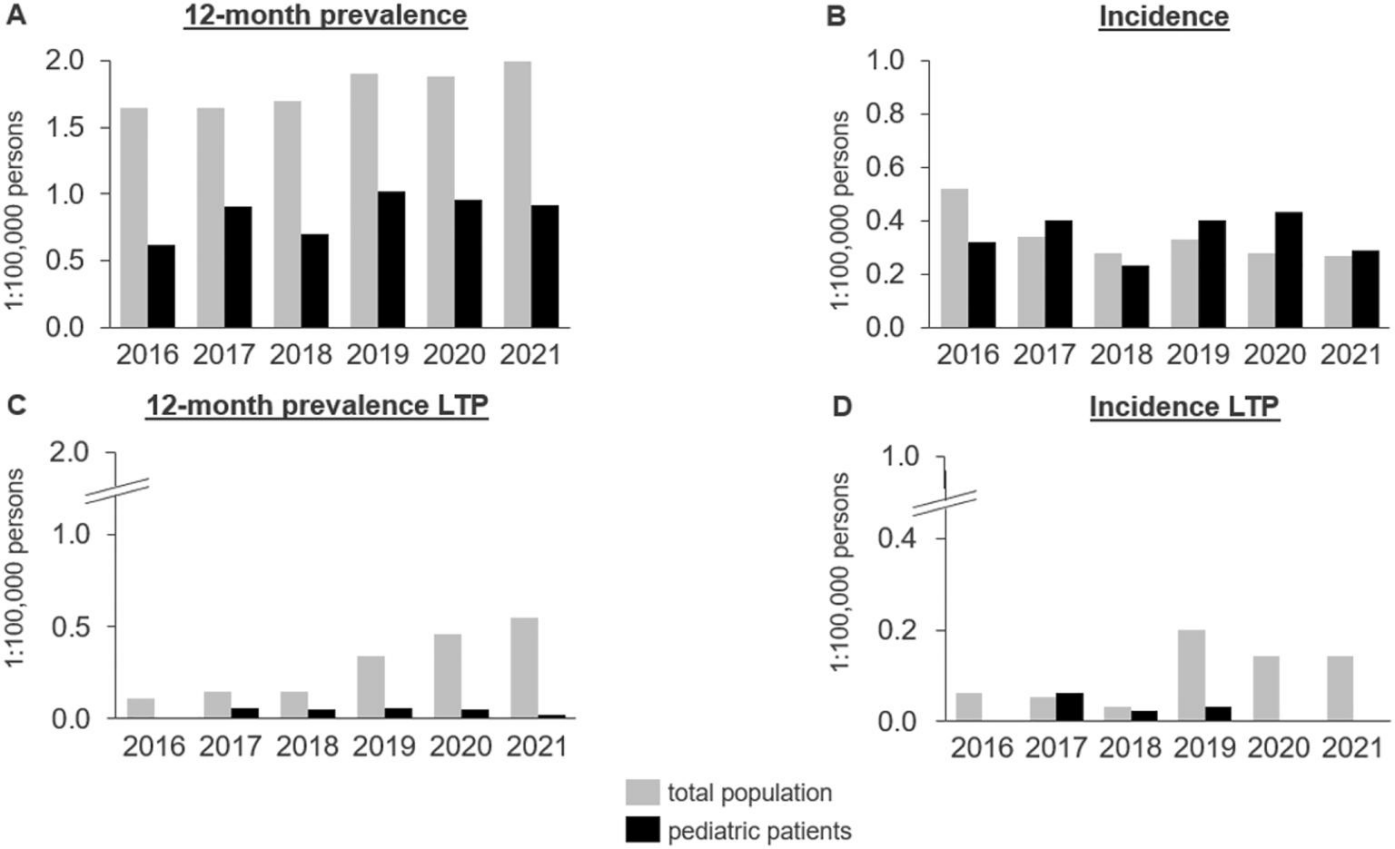
12‐month prevalence (A) and annual incidence (B) for HAE prescriptions overall and 12‐month prevalence (C) and annual incidence (D) for long‐term prophylaxis were obtained for the total population and pediatric patients.

The 12‐month prevalence ranged from 1.64:100,000 persons in 2016 and 2017 to 1.99:100,000 in 2021. Among children <12 years of age, the lowest 12‐month prevalence was observed in 2016 (0.62:100,000) and the highest in 2019 (1.02:100,000). The overall population incidence of 0.52:100,000 in 2016 was higher than that in subsequent years. Children <12 years had the highest incidence in 2020 at 0.43:100,000 persons (Figure 2).

In 2016–2021, the total number of SHI HAE patients with LTP was 498 (0.69:100,000 persons), including 8 children (0.11:100,000). The pediatric 12‐month prevalence increased from 0.11:100,000 in 2016 to 0.55:100,000 in 2021. In 2016, there were no pediatric patients <12 years with HAE‐LTP, and as of 2017, the annual prevalence was low (0.02–0.06:100,000). The incidence was highest in the total population in 2019 (0.20:100,000) and for pediatric patients in 2017 (0.06:100,000) (Figure 2, Supplementary Table S2). The results of the exploratory analysis of pediatric patients aged 2–11 years did not differ significantly from those of pediatric patients aged 0–11 (Supplementary Table S3).

### 4.2 Treating specialties of HAE patients

**FIGURE 3:**
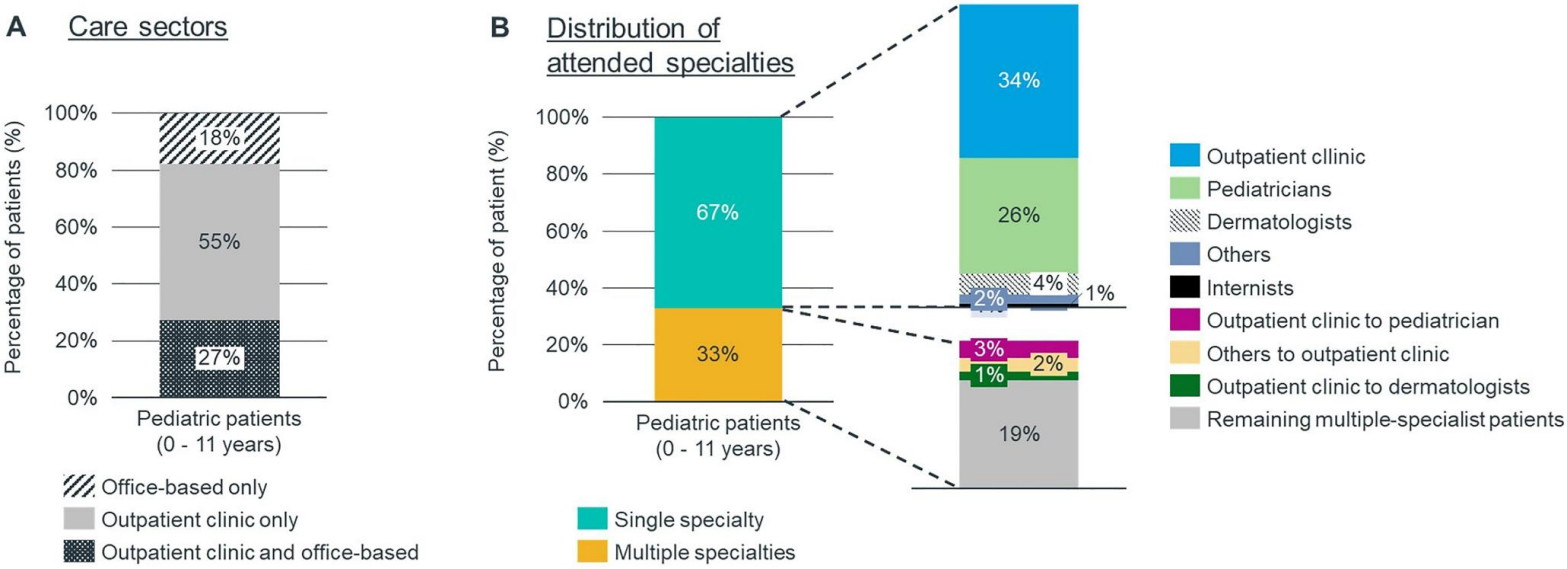
(A) For children (0–11 years), a breakdown was made by sector of care (no information on specialty). (B) For specialty analysis, patients were classified as having multiple or single specialists. For outpatient clinics with information on specialty, patients were classified as being treated by the appropriate specialty. Fifty‐nine percent of patients treated by pediatricians only were treated by office‐based physicians (further details in Supplementary Table S5).

Most pediatric patients received their HAE prescriptions exclusively from the outpatient sector (specialty not indicated), including university hospital outpatient departments (55%, Figure 3A). Most pediatric patients (<12 years) were treated in outpatient clinics (34%, no specialty recorded), followed by pediatricians (26%), of whom the majority (59%) treated their patients in an office‐based setting (Figure 3B). Moreover, 33% of pediatric patients received HAE prescriptions by multiple specialists, but the majority (67%) of patients remained with a single specialist throughout the observation period.

In the overall population, 70% of HAE patients were continuously treated by one specialty. Among these patients, 40% received prescriptions by outpatient clinics, followed by general practitioners (10%).

### 4.3 HAE treatments in children aged 2–11 years

**FIGURE 4:**
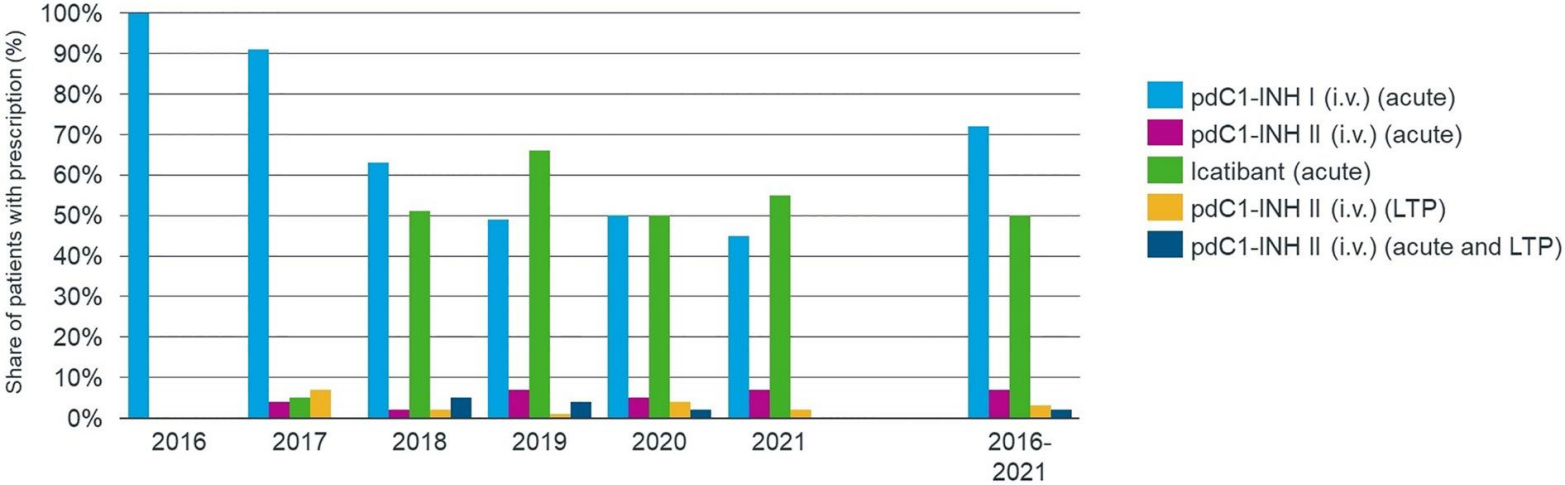
Multiple prescriptions of multiple substances per year were possible. For pdC1‐INH II (i.v.), on‐demand treatment was defined as <7 vials/28 days and long‐term prophylaxis (LTP) as ≥7 vials/28 days. Children presented as on‐demand treatment and LTP met both predefined criteria in the respective time periods.

Children aged 2–11 years received either intravenous pdC1‐INH I or II (on‐demand, LTP, or both) or icatibant. While the proportion receiving pdC1‐INH I (i.v.) decreased from 2016 to 2021 (from 100% to 45%), conversely, the proportion receiving icatibant increased over the study period (from 5% to 55%; Figure 4). In the subgroup of pediatric patients aged 2–5 years, no prescription of HAE‐LTP could be detected. Within this age group, only on‐demand HAE treatments were relevant, namely pdC1‐INH I/II (i.v.) and icatibant (Supplementary Table S6).

Especially in the 6‐ to 11‐year‐old patient group, a high rate of on‐demand HAE treatments could be observed. In particular, for pdC1‐INH I (i.v.), a vial usage of up to 190 vials per year was observed. For LTP, according to market authorization, only pdC1‐INH II (i.v.) was used throughout the study period. In patients aged 6–11 years, the highest consumption rate of pdC1‐INH II (i.v.) was 260 vials/year, which intriguingly declined to an average consumption of 160 vials/year by 2021 (Supplementary Table S6) and was also reflected in the average number of prescriptions (Supplementary Table S7).

While the consumption of vials of HAE‐on‐demand treatment in pediatric patients younger than 12 years remained high throughout the study period, the proportion of pediatric patients on both LTP and on‐demand treatment was low at 2%–6% (Supplementary Table S9).

### 4.4 Co‐medication

**FIGURE 5:**
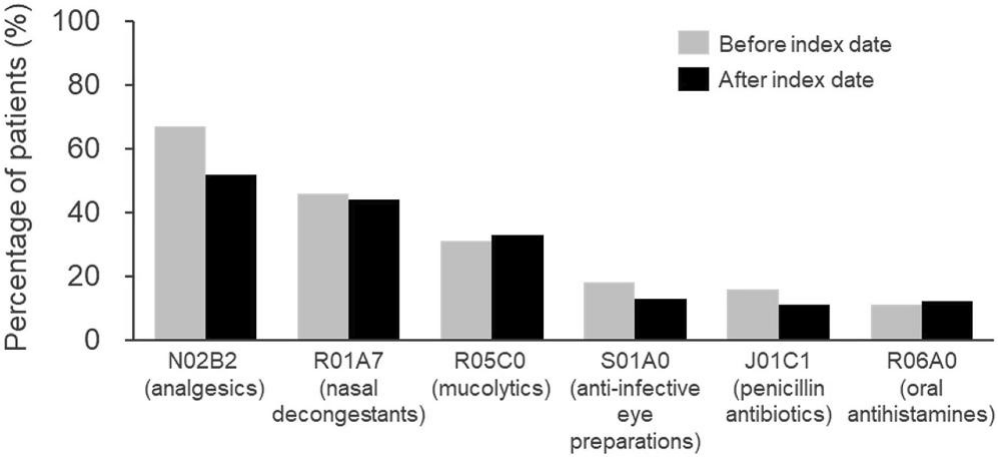
The index date is defined as the first prescription of HAE treatments between January 2016 and December 2020 for children (0–10 years).

The most frequently prescribed co‐medications in children with HAE were analgesics, with rates decreasing from 67% to 52% after the initial prescription of HAE treatment (index date) (Figure 5, Supplementary Table S10). Prescription rates for nasal decongestants (ATC: R01A7, 46% to 44%), expectorants (ATC: R05C0, 34% to 31%), and systemic antihistamines (ATC: R06A0, 11%–12%) did not differ substantially before and after the index date (Figure 5, Supplementary Table S10).

## 5 DISCUSSION

To our knowledge, this is the first study to provide detailed epidemiologic data of children with HAE based on real‐world evidence. For children aged <12 years, this study found a HAE prevalence of 2.51:100,000 over the study period (2016–2021, Figure 1), a 12‐month prevalence of up to 1.02:100,000, and an annual incidence up to 0.43:100,000 between 2016 and 2021. The outpatient clinic was identified as the most common institution for issuing prescriptions. The most frequently prescribed substances were human C1 inhibitor I (72%) and icatibant (50%) as on‐demand treatment and pdC1‐INH II (3%) as LTP (Figure 4, Supplementary Table S8).

The HAE prevalence in our total population was 3.35 per 100,000 persons, higher than the previously reported average data of 1‐2:100,000 persons but still within the reported range of 0.67–10:100,000.^2^, ^11^, ^12^, ^13^ The determined HAE 12‐month prevalence was 1.64–1.99:100,000, which was higher than previously shown in a survey‐based study from Austria (1.55:100,000).^14^ For children <12 years, the 12‐month prevalence ranged from 0.62 to 1.02:100,000 in the study, which is in the same range as published data from a survey in the UK (1: 100,000).^15^ The increased prevalence may be due to the nature of the study. Our pharmacy claims‐based study covered the vast majority of the population and thus serves as a reliable real‐world data source, which is particularly important for rare and frequently underdiagnosed diseases and may explain higher numbers in prevalence compared to other sources. In contrast, surveys or registries may underestimate epidemiologic data if not all relevant sources are considered or not all disease cases are reported.^16^, ^17^ The prevalence of HAE was lower in pediatric patients, consistent with the fact that some patients are not recognized until adolescence with a reported diagnostic delay of up to 15 years.^14^ However, it is important to reduce this delay in order to initiate specific HAE treatment with the aim to reduce the burden of the disease and the risk of fatal outcomes.^3^, ^6^, ^18^, ^19^ The incidence was slightly higher in pediatric patients compared with the overall population, which may reflect a distinct proportion of pediatric patients with a known family history. Conversely, without a positive family history, the diagnosis of HAE can be challenging and thus may be delayed.^20^

The analysis of the treating specialties is relevant for future clinical studies as well as for the awareness and knowledge transfer to relevant healthcare professionals as recommended using the international HAE guidelines and the international HAE patient organization HAEi.^3^ Moreover, knowledge of treating specialties is important as continuity of care is crucial for pediatric patients with rare and complex conditions.^21^ Thus, the fact that two‐thirds of the pediatric patients were treated by only one specialty appears favorable, with outpatient clinics and pediatricians as the main prescribers. In the total population, the spectrum of disciplines was more diverse, which is well known from the literature but contradicts the guideline's recommendation to mainly provide care through comprehensive angioedema centers.^3^

In Germany, HAE centers are almost exclusively located at university hospitals, which could explain the significantly high proportion of outpatient clinics and may, at the same time, have contributed to a small number of patient journeys.^22^, ^23^ However, the HAE centers could not be identified within the outpatient sector in the current study as they are not specifically classified in the LRx database.

The prescription data of the present study reflected the approval status of HAE treatments. For on‐demand treatment, there was a significant increase in prescriptions for icatibant, consistent with the label extension at the end of 2017 for pediatric patients aged ≥2 years. Although home‐based therapy is well established in HAE care and self‐administration has been shown to be feasible and safe for school‐aged pediatric patients, there are challenges with intravenous use of pdC1‐INH, particularly in young children.^24^, ^25^, ^26^ The ease of use with ready‐to‐use, subcutaneous administration of icatibant as well as patient preferences may have played a role in the increase of icatibant usage and the decrease of pdC1‐INH consumption.^27^, ^28^

In contrast to the development of icatibant, conestat alfa was not prescribed in children aged 2–11 years despite approval in Germany in May 2020. This may be due to the limited data available, particularly for preschoolers, and the less cost‐effective dose of active ingredient per vial for this age group.^28^ In 2016 and 2017, a high number of pdC1‐INH I vials were prescribed in the ≥6 years age group (159 and 190 vials, Supplementary Table S6), which was interestingly not associated with an increased incidence. The lower number in subsequent years may have been caused by the expansion of the pediatric approval of icatibant, which offered a markedly reduced burden of treatment. However, there was an overall increase in prescriptions for HAE medication in pediatric patients in each subsequent year of treatment. In addition to a necessary dose adjustment with increasing body weight, this might be explained by an increase in HAE disease activity and attacks towards puberty, while simultaneously a low LTP treatment rate was observed.^29^ Higher consumption of on‐demand HAE treatments without higher LTP rates could indicate a need for more convenient pediatric LTP options.

In our total population, the proportion of patients with HAE LTP treatment ranged from 7.0% (2016) to 27.9% (2021). The LTP treatment rate of an international registry was slightly higher at 30%, and the rates of a recent French study^30^ and those in the United Kingdom HAE registry were significantly higher (59.2% and 45%, respectively).^31^, ^32^ However, a large proportion of these patients were treated with androgens and progestins, which carry side effect risks and are not approved for HAE in Germany.^33^

The highest LTP treatment rate in children ≥6 years observed in our study was 7% (*n* = 4) in 2017; however, this decreased to 0 in 2021 (Supplementary Table S8). The fact that there was no documented LTP in children 2–5 years of age and a declining rate in children 6–11 years of age may indicate an unmet need for better LTP options in this age group. This is consistent with registry data in the United Kingdom with an LTP rate in children younger than 12 years of 6% versus 53% in adults.^32^ The proportion of pediatric patients (<18 years) with LTP in the global HAE registry was 8.6%, two‐thirds of whom received tranexamic acid, which was not included in our study.^31^ This could be due to the high treatment burden for children under 6 years of age as there are currently only intravenous LTP options available. Intravenous administration in younger children can be much more difficult due to anxiety and more challenging venous access. This fact potentially makes it difficult for children and/or parents to ensure compliance with LTP and could encourage re‐switches to acute treatment if the disease burden is not too severe. Potentially excluded LTP patients would still be captured in our analyses via the analysis of the additional use of on‐demand treatments.

Following the approval of lanadelumab and pdC1‐INH I (s.c.) as HAE‐LTP for patients ≥12 years in 2018, the incidence of HAE‐LTP in the total population increased from 2019. In contrast, after the approval of berotralstat in mid‐2021 for patients ≥12 years, the incidence remained unaffected. For children <12 years of age, no off‐label prescriptions for lanadelumab, pdC1‐INH I (s.c.), and berotralstat were observed. In 2017, pdC1‐INH II (i.v.) received a label extension for LTP in pediatric patients ≥6 years, which initially led to an increase in LTP incidence and prevalence but was followed by a steep decline in subsequent years (Supplementary Table S2). Given the concomitant increase in on‐demand HAE treatments in pediatric patients aged 6–11 years, there may be an unmet need for newer, improved LTP treatment options without frequent intravenous injections. This is supported by a U.S. study based on pharmacy claims, in which a 53% proportion of emergency treatments has been interpreted as an unmet need for effective LTP.^34^

Analysis of co‐medication showed a significant decrease in analgesic prescriptions (non‐steroidal anti‐inflammatory drugs) after HAE diagnosis. The most commonly prescribed drug in this group (ATC code N02B) was acetaminophen/paracetamol. This may be related to the natural decrease in febrile respiratory infections during childhood, but also to the decrease in colicky abdominal pain due to HAE‐specific treatment.^35^ The latter is supported by the fact that no relevant differences were found for expectorants and nasal decongestants. Even though HAE is often misdiagnosed as mast cell‐mediated angioedema, the number of prescriptions for systemic antihistamines remained unchanged, which may be due to the general high prevalence of atopic diseases.^3^, ^8^ Correct clinical interpretation of swellings may also have influenced the decrease in oral antibiotics.

Using a database of pharmacy claims for disease‐specific treatments, we were able to generate population‐based data for HAE. However, several limitations must be considered when interpreting the presented results. The LRx database does currently cover 84% of all prescriptions reimbursed in Germany. Not all pharmacy collection centers deliver their data for analysis. Since prescription data lack information on diagnoses and associated laboratory tests, treatment prescriptions are used as surrogate markers for diagnoses. While patients without prescriptions and inpatient therapies are not recorded, those with unconfirmed diagnoses will be recorded if a prescription is made. In addition, it is not possible to obtain details on familial relationships or mutation frequency as this information is not included or accessible in the LRx database. Furthermore, although the use of tranexamic acid in children is uncommon in Germany and not recommended in consensus statements, pediatric patients with tranexamic acid as LTP may have been missed due to the inclusion criteria of our analysis.^8^

Our data cannot distinguish whether a treatment was actually administered or held back by the patient for a possible severe attack. The assignment of pdC1‐INH II (i.v.) as an on‐demand treatment or LTP was derived from assumptions about vial consumption (>7 vials/28 days) for pediatric patients and adults alike. For individual patients, the month of birth was not available and was set to January of the year of birth, which may have resulted in a minor overestimation of age.

Additionally, a minor overestimation of incidence in 2016 may have resulted from a slightly prolonged lookback period in the previous year (Figure 1).

In summary, our study found an HAE prevalence in children <12 years of 2.51:100,000 and a 12‐month prevalence of up to 1.02:100,000, which is higher than the previously reported average. Outpatient clinics and pediatricians were the main prescribers of HAE treatments, which comprised an increasing proportion of icatibant as on‐demand treatment. The rate of LTP treatment was low, which may highlight an unmet‐need for newer, improved HAE treatment options in childhood.

## AUTHOR CONTRIBUTIONS

**Freerk Prenzel**: Conceptualization (equal); data curation (equal); methodology (equal); supervision (equal); validation (equal); writing—review and editing (equal). **Susanne Abraham**: Conceptualization (equal); data curation (equal); methodology (equal); supervision (equal); validation (equal); writing—review and editing (equal). **Christoph Hirche**: Conceptualization (equal); data curation (equal); formal analysis (equal); methodology (equal); validation (equal); writing—original draft (equal); writing—review and editing (equal). **Gerrit Müller**: Conceptualization (equal); data curation (equal); formal analysis (equal); methodology (equal); validation (equal); writing—original draft (equal); writing—review and editing (equal). **Stephan Kaiser**: Conceptualization (equal); formal analysis (equal); investigation (equal); methodology (equal); project administration (equal); writing—review and editing (equal). **Leonarda Serdani‐Neuhaus**: Data curation (equal); project administration (equal); visualization (equal); writing—original draft (equal); writing—review and editing (equal). **Rebecca Zingel**: Conceptualization (equal); formal analysis (equal); investigation (equal); software (equal); visualization (equal); writing—original draft (equal); writing—review and editing (equal). **Inmaculada Martinez Saguer**: Conceptualization (equal); data curation (equal); methodology (equal); supervision (equal); validation (equal); writing—review and editing (equal).

## CONFLICT OF INTEREST STATEMENT

The authors declare that they have no relevant or material financial interests that relate to the data and research described in this publication. Freerk Prenzel received honoraria, research funding, and travel grants from Sanofi Genzyme, Novartis, Leipziger Gesundheitsnetz, Berlin‐Chemie, Allergopharma, Vertex, Nutricia‐Milupa, Takeda, medilearn and Stallergenes Greer. Susanne Abraham has received speaking and/or consulting fees and is involved in clinical trials for Novartis, Sanofi, Beiersdorf, UCB, Amgen, LEO Pharma, Takeda, Lilly, Boehringer Ingelheim, and AbbVie. Inmaculada Martinez‐Saguer has received honoraria, research funding, and travel grants from BioCryst, CSL Behring, Pharming, Octapharma, KalVista, andTakeda/Shire and/or has served as a consultant and/or participated in advisory boards for these companies. Christoph Hirche, Gerrit Müller, Stephan Kaiser and Leonarda Serdani‐Neuhaus are employees of Takeda Pharma Vertrieb GmbH and Co. KG. Rebecca Zingel is an employee of IQVIA, Frankfurt Am Main, Germany, who conducted the analysis.

## Supporting information

Supporting Information S1

Supporting Information S2

## Data Availability

The data that support the findings of this study are available on request from the corresponding author. The data are not publicly available due to privacy or ethical restrictions.
