# Supplementary material for: Epidemiology and treatment of children with hereditary angioedema in Germany: A retrospective database study
Source: Clin Transl Allergy. 2023 Nov 13;13(11):e12313. doi: 10.1002/clt2.12313 (PMC10642561; doi:10.1002/clt2.12313)

**x. Appendices**

**STUDY SUPPLEMENT**

Table S1: Statutory health insurance population as basis for prevalence and incidence

|  | **2016** | **2017** | **2018** | **2019** | **2020** | **2021** | **Total (mean)** |
| --- | --- | --- | --- | --- | --- | --- | --- |
| SHI total population | | | | | | | |
| Total | 71 449 138 | 72 258 037 | 72 802 098 | 73 009 237 | 73 357 859 | 73 318 711 | 72 699 180 |
| SHI pediatric population | | | | | | | |
| # SHI population (0 -14 years) | 9 047 366 | 9 296 278 | 9 424 220 | 9 537 719 | 9 659 855 | 9 729 727 | 9 449 194 |
| # children (0 - 14 years) | 11 048 568 | 11 171 759 | 11 290 815 | 11 391 259 | 11 477 800 | 11 477 800 (proxy) | 11 309 667 |
| Share of SHI population (0 - 14 years) | 82% | 83% | 83% | 84% | 84% | 85% | 84% |
| # children (0 - 11 years) | 8 810 951 | 8 946 821 | 9 076 510 | 9 172 385 | 9 236 880 | 9 236 880 (proxy) | 9 080 071 |
| Estimated # of SHI population (0 - 11 years) | 7 215 043 | 7 444 856 | 7 575 983 | 7 679 891 | 7 773 870 | 7 830 100 | 7 586 642 |
| # children (2-11 years) | 7 260 133 | 7 365 598 | 7 496 158 | 7 603 383 | 7 683 907 | 7 683 907 (proxy) | 7 515 514 |
| Estimated # of SHI population (2-11 years) | 5 945 122 | 6 129 084 | 6 256 895 | 6 366 191 | 6 466 869 | 6 513 645 | 6 279 634 |

Table S2: HAE 12-month prevalence, annual incidence, and long-term prophylaxis in the total study population and in pediatric patients aged 0-11 years per 100,000 persons.

| **Year** | **study group** | **12-month prevalence** | **12-month incidence** | **12-month LTP prevalence** | **12-month LTP incidence** |
| --- | --- | --- | --- | --- | --- |
| 2016 | total population | 1.64 | 0.52 | 0.11 | 0.06 |
|  | pediatric patients | 0.62 | 0.32 | 0.00 | 0.00 |
| 2017 | total population | 1.64 | 0.34 | 0.15 | 0.05 |
|  | pediatric patients | 0.91 | 0.40 | 0.06 | 0.06 |
| 2018 | total population | 1.70 | 0.28 | 0.15 | 0.03 |
|  | pediatric patients | 0.70 | 0.23 | 0.05 | 0.02 |
| 2019 | total population | 1.90 | 0.33 | 0.34 | 0.20 |
|  | pediatric patients | 1.02 | 0.40 | 0.06 | 0.03 |
| 2020 | total population | 1.88 | 0.28 | 0.46 | 0.14 |
|  | pediatric patients | 0.96 | 0.43 | 0.05 | 0.00 |
| 2021 | total population | 1.99 | 0.27 | 0.55 | 0.14 |
|  | pediatric patients | 0.92 | 0.29 | 0.02 | 0.00 |

Table S3: Exploratory analysis of 12-month prevalence and annual incidence of pediatric patients (2-11 years) on HAE treatment and on long-term HAE prophylaxis

| **Year** | **Study group** | **Estimated 12-month prevalence HAE (in 1:100,000)** | **Estimated incidence HAE (in 1:100,000)** | **Estimated 12-month prevalence HAE LTP (in 1:100,000)** | **Estimated incidence HAE LTP (in 1:100,000)** |
| --- | --- | --- | --- | --- | --- |
| 2016 | pediatric patients  (2-11 years) | 0.68 | 0.31 | 0.00 | 0.00 |
| 2017 | pediatric patients  (2-11 years) | 1.07 | 0.45 | 0.08 | 0.08 |
| 2018 | pediatric patients  (2-11 years) | 0.76 | 0.19 | 0.06 | 0.02 |
| 2019 | pediatric patients  (2-11 years) | 1.21 | 0.47 | 0.07 | 0.04 |
| 2020 | pediatric patients  (2-11 years) | 1.03 | 0.37 | 0.06 | 0.00 |
| 2021 | pediatric patients  (2-11 years) | 1.07 | 0.29 | 0.02 | 0.00 |

Table S4: Prevalence in the total population and among pediatric patients stratified by region and gender

|  | **Share of patients** | **Estimated prevalence (in 1:100,000)** |
| --- | --- | --- |
| Total population (n= 2,082) | | |
| Gender | | |
| Female | 66.2% | 3.91 |
| Male | 33.8% | 2.16 |
| Region | | |
| East | 16.7% | 2.73 |
| North | 7.6% | 3.29 |
| South | 22.0% | 3.99 |
| West | 53.6% | 4.35 |
| Pediatric patients aged 0-11 years (n=163) | | |
| Gender | | |
| Female | 55.5% | 2.41 |
| Male | 44.5% | 1.83 |
| Region | | |
| East | 20.9% | 2.60 |
| North | 6.1% | 2.01 |
| South | 17.8% | 2.39 |
| West | 55.2% | 3.35 |
| Pediatric patients aged 2-11 years (n=139) | | |
| Gender | | |
| Female | 56.3% | 2.57 |
| Male | 43.7% | 1.89 |
| Region | | |
| East | 20.9% | 2.66 |
| North | 6.5% | 2.19 |
| South | 15.8% | 2.20 |
| West | 56.8% | 3.56 |

Table S5. Incident patients with HAE treated by a single specialist

| **Treating specialty**  **(0- 11 years)** | **Patient N in LRx (N = 135) (patient share)** | **Treating specialty**  **(Total population)** | **Patient N in LRx (N = 1,255) (patient share)** |
| --- | --- | --- | --- |
| Outpatient clinic | 46 (34%) | Outpatient clinic | 503 (40%) |
| Pediatricians | 35 (26%) | General practitioner | 120 (10%) |
| Dermatologists | 6 (4%) | Pediatricians | 106 (8%) |
| Others | 3 (2%) | Dermatologists | 88 (7%) |
| Internal specialists | <3 (1%) | Others | 55 (4%) |
|  |  | Internal specialists | 17 (1%) |

**Table S6: The average number of vials prescribed per substance per year and PCN (pharmaceutical central number) for pediatric patients**

| **Type of treatment** | **Substance** | **Time period** | | | | | |
| --- | --- | --- | --- | --- | --- | --- | --- |
| **2-5 years** | | **2016** | **2017** | **2018** | **2019** | **2020** | **2021** |
| On-demand treatment | pdC1-INH I (i.v.) - 1 vial/pack (PCN 1) | 5.4 | 5.1 | 10.6 | 12.5 | 7.9 | 10.4 |
|  | pdC1-INH I (i.v.) - 1 vial/pack (PCN 3) | *n.a.* | | | | | 1.0 |
|  | pdC1-INH II (i.v.) - 2 vials/pack (PCN 2) | *n.a.* | | 22.0 | 41.0 | 0.0 | 20.0 |
|  | Icatibant - 1 vial/pack (PCN 1) | *n.a.* | 1.0 | 1.1 | 1.3 | 1.3 | 1.7 |
|  | Icatibant - 3 vials/pack (PCN 2) | *n.a.* | | 3.0 | 3.0 | 4.5 | 4.0 |
|  | Icatibant - 3 vials/pack (PCN 4) | *n.a.* | | | | | 3.0 |
| **6-11 years** | | **2016** | **2017** | **2018** | **2019** | **2020** | **2021** |
| On-demand treatment | pdC1-INH I (i.v.) - 1 vial/pack (PCN 1) | 16.1 | 17.8 | 27.7 | 19.7 | 17.9 | 18.8 |
|  | pdC1-INH I (i.v.) - 1 vial/pack (PCN 2) | 159.0 | 190.0 | 28.0 | 10.0 | 8.7 | 9.0 |
|  | pdC1-INH II (i.v.) - 2 vials/pack (PCN 2) | *n.a*. | 20.0 | *n.a.* | | | |
|  | pdC1-INH II (i.v.) - 2 vials/pack (PCN 2) | *n.a.* | | | 4.7 | 17.3 | 8.0 |
|  | Icatibant - 1 vial/pack (PCN 1) | *n.a.* | 1.5 | 1.8 | 1.3 | 2.0 | 1.3 |
|  | Icatibant - 3 vials/pack (PCN 2) | *n.a.* | 3.0 | 7.0 | 7.9 | 5.3 | 6.3 |
|  | Icatibant - 3 vials/pack (PCN 3) | *n.a.* | | | | | 6.0 |
|  | Icatibant - 3 vials/pack (PCN 4) | *n.a.* | | | | | 3.0 |
| LTP | pdC1-INH II (i.v.) - 2 vials/pack (PCN 1) | *n.a.* | 92.5 | *n.a.* | | | |
|  | pdC1-INH II (i.v.) - 2 vials/pack (PCN 2) | *n.a.* | | 40.0 | 260.0 | 160.0 | 160.0 |
| On-demand treatment and LTP | pdC1-INH II (i.v.) - 2 vials/pack (PCN 2) | *n.a.* | | 100.0 | 105.3 | 200.0 | *n.a.* |

Table S7: Average number of prescriptions per treatment and per calendar or treatment year in pediatric patients (2-11 years)

| **Type of treatment** | **Compound** | **Time period** | | | | | |
| --- | --- | --- | --- | --- | --- | --- | --- |
| **By calendar year** | | **2016** | **2017** | **2018** | **2019** | **2020** | **2021** |
| On-demand treatment | pdC1-INH I (i.v.) | 2.7 | 2.0 | 2.8 | 2.4 | 1.9 | 2.0 |
|  | pdC1-INH II (i.v.) | *n.a.* | 1.5 | 3.0 | 1.6 | 2.0 | 1.3 |
|  | Icatibant | *n.a.* | 1.3 | 1.7 | 1.5 | 1.5 | 1.3 |
| LTP | pdC1-INH II (i.v.) | *n.a.* | 2.8 | 1.0 | 7.0 | 4.5 | 4.0 |
| On-demand treatment and LTP | pdC1-INH II (i.v.) | *n.a.* | | 7.0 | 5.7 | 7.0 | 0.0 |
| **By treating year** | | **1^st^ treating year** | | **2^nd^ treating year** | **3^rd^ treating year** | **4^th^ treating year** | **5^th^ treating year** |
| On-demand treatment | pdC1-INH I (i.v.) | 1.9 | | 3.6 | 2.6 | 3.1 | 3.0 |
|  | pdC1-INH II (i.v.) | 1.7 | | 4.0 | 3.0 | *n.a.* | |
|  | Icatibant | 1.5 | | 1.2 | 2.1 | 1.4 | 2.0 |
| LTP | pdC1-INH II (i.v.) | 2.0 | | 2.0 | 4.0 | 3.5 | 5.0 |
| On-demand treatment and LTP | pdC1-INH II (i.v.) | *n.a.* | | 4.7 | 6.5 | 7.0 | 6.0 |

Table S8: Number of pediatric patients (2-11 years) for each HAE treatment overall and annually.

| **Time period** | **Type of treatment** | **Compound** | **Patient N in LRx (patient share)** |
| --- | --- | --- | --- |
| 2016 - 2021  (N LRx patients total = 153) | On-demand treatment | pdC1-INH I (i.v.) | 110 (72%) |
|  |  | pdC1-INH II (i.v.) | 11 (7%) |
|  |  | Icatibant | 77 (50%) |
|  |  | Conestat alfa | 0 (0%) |
|  | LTP | pdC1-INH I (s.c.) | 0 (0%) |
|  |  | pdC1-INH II (i.v.) | 4 (3%) |
|  |  | Berotralstat | 0 (0%) |
|  |  | Lanadelumab | 0 (0%) |
|  | On-demand treatment and LTP | pdC1-INH II (i.v.) | 3 (2%) |
| 2016  (N LRx patients total = 35) | On-demand treatment | pdC1-INH I (i.v.) | 35 (100%) |
|  |  | pdC1-INH II (i.v.) | 0 (0%) |
|  |  | Icatibant | 0 (0%) |
|  |  | Conestat alfa | 0 (0%) |
|  | LTP | pdC1-INH I (s.c.) | 0 (0%) |
|  |  | pdC1-INH II (i.v.) | 0 (0%) |
|  |  | Berotralstat | 0 (0%) |
|  |  | Lanadelumab | 0 (0%) |
|  | On-demand treatment and LTP | pdC1-INH II (i.v.) | 0 (0%) |
| 2017  (N LRx patients total = 57) | On-demand treatment | pdC1-INH I (i.v.) | 52 (91%) |
|  |  | pdC1-INH II (i.v.) | <3 (4%) |
|  |  | Icatibant | 3 (5%) |
|  |  | Conestat alfa | 0 (0%) |
|  | LTP | pdC1-INH I (s.c.) | 0 (0%) |
|  |  | pdC1-INH II (i.v.) | 4 (7%) |
|  |  | Berotralstat | 0 (0%) |
|  |  | Lanadelumab | 0 (0%) |
|  | On-demand treatment and LTP | pdC1-INH II (i.v.) | 0 (0%) |
| 2018  (N LRx patients total = 41) | On-demand treatment | pdC1-INH I (i.v.) | 26 (63%) |
|  |  | pdC1-INH II (i.v.) | <3 (2%) |
|  |  | Icatibant | 21 (51%) |
|  |  | Conestat alfa | 0 (0%) |
|  | LTP | pdC1-INH I (s.c.) | 0 (0%) |
|  |  | pdC1-INH II (i.v.) | <3 (2%) |
|  |  | Berotralstat | 0 (0%) |
|  |  | Lanadelumab | 0 (0%) |
|  | On-demand treatment and LTP | pdC1-INH II (i.v.) | <3 (5%) |
| 2019  (N LRx patients total = 67) | On-demand treatment | pdC1-INH I (i.v.) | 33 (49%) |
|  |  | pdC1-INH II (i.v.) | 5 (7%) |
|  |  | Icatibant | 44 (66%) |
|  |  | Conestat alfa | 0 (0%) |
|  | LTP | pdC1-INH I (s.c.) | 0 (0%) |
|  |  | pdC1-INH II (i.v.) | <3 (1%) |
|  |  | Berotralstat | 0 (0%) |
|  |  | Lanadelumab | 0 (0%) |
|  | On-demand treatment and LTP | pdC1-INH II (i.v.) | 3 (4%) |
| 2020  (N LRx patients total = 56) | On-demand treatment | pdC1-INH I (i.v.) | 28 (50%) |
|  |  | pdC1-INH II (i.v.) | 3 (5%) |
|  |  | Icatibant | 28 (50%) |
|  |  | Conestat alfa | 0 (0%) |
|  | LTP | pdC1-INH I (s.c.) | 0 (0%) |
|  |  | pdC1-INH II (i.v.) | <3 (4%) |
|  |  | Berotralstat | 0 (0%) |
|  |  | Lanadelumab | 0 (0%) |
|  | On-demand treatment and LTP | pdC1-INH II (i.v.) | <3 (2%) |
| 2021  (N LRx patients total = 58) | On-demand treatment | pdC1-INH I (i.v.) | 26 (45%) |
|  |  | pdC1-INH II (i.v.) | 4 (7%) |
|  |  | Icatibant | 32 (55%) |
|  |  | Conestat alfa | 0 (0%) |
|  | LTP | pdC1-INH I (s.c.) | 0 (0%) |
|  |  | pdC1-INH II (i.v.) | <3 (2%) |
|  |  | Berotralstat | 0 (0%) |
|  |  | Lanadelumab | 0 (0%) |
|  | A On-demand treatment and LTP | pdC1-INH II (i.v.) | 0 (0%) |

Table S9: Share of pediatric patients (2-11 years, n=153) on long-term prophylaxis in addition to on-demand treatment

| **Year** | **LRx Patients with on-demand treatment** | **Thereof LRx patients  with LTP (patient share)** |
| --- | --- | --- |
| 2016 | 35 | 0 (0%) |
| 2017 | 55 | <3 (4%) |
| 2018 | 40 | <3 (5%) |
| 2019 | 67 | 4 (6%) |
| 2020 | 54 | <3 (2%) |
| 2021 | 57 | 0 (0%) |

Table S10: Co-medication within one year before and after the index date of HAE treatment in children aged 0-10 years (n=103)

| **Before the index date** | | **After the index date** | |
| --- | --- | --- | --- |
| **EphMRA ATC class** | **Patient N in LRx (patient share)** | **EphMRA ATC class** | **Patient N in LRx (patient share)** |
| N02B2 – Analgesics | 69 (67%) | N02B2 – Analgesics | 54 (52%) |
| R01A7 – Nasal decongestants | 47 (46%) | R01A7 – Nasal decongestants | 45 (44%) |
| R05C0 - Expectorants | 32 (31%) | R05C0 - Expectorants | 34 (33%) |
| S01A0 – Anti-infectives-eye | 19 (18%) | S01A0 – Anti-infectives-eye | 13 (13%) |
| J01C1 – Broad spectrum penicillins oral | 16 (16%) | R06A0 –Antihistamines systemic | 12 (12%) |
| R03A4 – Short-acting B2-agonists inhalant | 16 (16%) | D07A0 – Topical corticosteroids plain | 12 (12%) |
| A11C2 – Vitamin D | 14 (14%) | J01C1 – Broad spectrum penicillins oral | 11 (11%) |
| D07A0 – Topical corticosteroids plain | 12 (12%) | A01A0 – Stomatologicals | 10 (10%) |
| R06A0 – Antihistamines systemic | 11 (11%) | A07F0 – Antidiarrhoeal micro-organisms | 8 (8%) |
| A04A9 – Other antiemetics & antinausea | 11 (11%) | J01D1 – Cephalosporins oral | 8 (8%) |
|  |  | R03A4 – Short-acting B2-agonists inhalant | 8 (8%) |

Figure F1: Regional prevalence in the total population (n=2.082) and in children aged 0-11 years (n=163) (2016 - 2021)


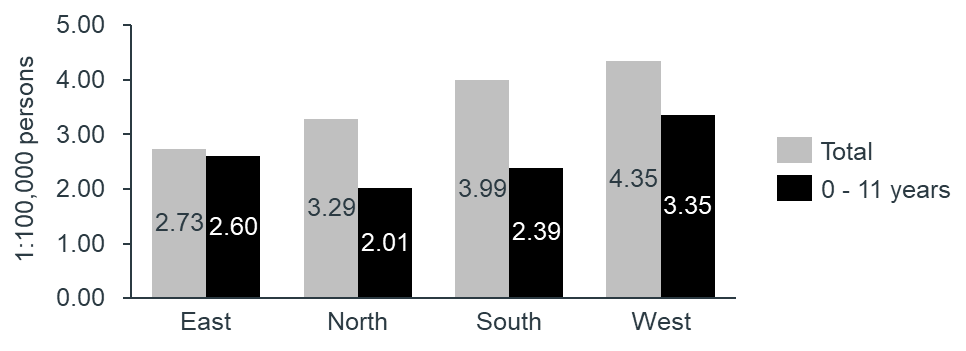

Supplement: Supplementary file 1 — Supporting Information S1 [file CLT2-13-e12313-s001.docx]
