# Supplementary material for: Epidemiology and treatment of children with hereditary angioedema in Germany: A retrospective database study
Source: Clin Transl Allergy. 2023 Nov 13;13(11):e12313. doi: 10.1002/clt2.12313 (PMC10642561; doi:10.1002/clt2.12313)
Supplement: Supplementary file 2 — Supporting Information S2 [file CLT2-13-e12313-s002.docx]

Supplement Legends

Table S1: Number of statutory health insured population in all subgroups (total population, pediatric patients: 0-11 and 2-11 years (exploratory analysis)) deducted from DESTATIS and German Federal Health Reporting system.

Table S2: HAE and LTP 12-month prevalence and annual incidence in the total population and pediatric patients (data shown in Figure 2).

Table S3: Exploratory analysis in the pediatric patients aged 2-11 years.

Table S4: Estimated prevalence (1:100,000) in the total population and pediatric patients aged 0-11 and 2-11 years (exploratory analysis) from 2016 to 2021.

Table S5: Share of treating specialties in the total population and in pediatric patients (0-11 years, Figure 3).

Table S6: Annual average vial prescriptions in pediatric patients aged 2-5 and 6-11 years (2016-2021).

Depicted as not applicable (n.a.), if either treatment was not detected or fewer than 3 patients were detected during the relevant time period. In the group of pediatric patients aged 2-5 years, no LTP/on-demand or LTP treatments could be detected in the study cohort. PdC1-INH II (i.v.) on-demand treatment was defined as <7 vials/28 days and LTP as ≥7 vials/28 days. Pediatric patients depicted as on-demand treatment and LTP met both pre-defined criteria in the respective time period.

Table S7: Annual average number of prescriptions in pediatric patients aged 2-11 years (2016-2021).

Depicted as not applicable (n.a.), if either treatment was not detected or fewer than 3 patients were detected during the relevant time period. In the group of pediatric patients aged 2-5 years, no LTP/on-demand or LTP treatments could be detected in the study cohort. PdC1-INH II (i.v.) on-demand treatment was defined as <7 vials/28 days and LTP as ≥7 vials/28 days. Pediatric patients depicted as on-demand treatment and LTP met both pre-defined criteria in the respective time period.

Table S8: Share of therapies in pediatric patients (2-11 years).

PdC1-INH II (i.v.) on-demand treatment was defined as <7 vials/28 days and LTP as ≥7 vials/28 days. Pediatric patients depicted as on-demand treatment and LTP met both pre-defined criteria in the respective time period.

Table S9: Share of pediatric patients (2-11 years) with both on-demand and LTP treatment.

Table S10: Co-medication one year before and after first HAE prescription (0-10 years), categorized according to EphMRA ATC classes.

Figure F1: Prevalence in the total population and in pediatric patients stratified by region.
